# Novel dual LSD1/HDAC6 inhibitor for the treatment of cancer

**DOI:** 10.1371/journal.pone.0279063

**Published:** 2023-01-03

**Authors:** Chandru Gajendran, Subramanyam Janardhan Tantry, Naveen Sadhu M., Zainuddin Mohammed, Purushottam Dewang, Mahanandeesha Hallur, Sreekala Nair, Krishnakumar Vaithilingam, Basavaprabhu Nagayya, Sridharan Rajagopal, Dhanalakshmi Sivanandhan

**Affiliations:** 1 Jubilant Therapeutics India Ltd, Bangalore, India; 2 Department of Medicinal Chemistry, Jubilant Biosys Ltd, Bangalore, India; 3 Department of Discovery Biology, Jubilant Biosys Ltd, Bangalore, India; 4 Department of Structural Biology, Jubilant Biosys Ltd, Bangalore, India; Ludwig-Maximilians-Universitat Munchen Adolf-Butenandt-Institut, GERMANY

## Abstract

Dually targeting the epigenetic proteins lysine specific demethylase 1 (LSD1) and histone deacetylases (HDACs) that play a key role in cancer cells by modulating gene repressor complexes including CoREST will have a profound effect in inhibiting tumour growth. Here, we evaluated JBI-097 a dual LSD1/HDAC6 inhibitor, for its *in vitro* and *in vivo* activities in various tumor models. *In vitro*, JBI-097 showed a strong potency in inhibiting LSD1 and HDAC6 enzymatic activities with the isoform selectivity over other HDACs. Cell-based experiments demonstrated a superior anti-proliferative profile against haematological and solid tumor cell lines. JBI-097 also showed strong modulation of HDAC6 and LSD1 specific biomarkers, alpha-tubulin, CD86, CD11b, and GFi1b. *In vivo*, JBI-097 showed a stronger effect in erythroleukemia, multiple myeloma xenograft models, and in CT-26 syngeneic model. JBI-097 also showed efficacy as monotherapy and additive or synergistic efficacy in combination with the standard of care or with immune checkpoint inhibitors. These and other findings suggest that JBI-097 could be a promising molecule for targeting the LSD1 and HDAC6. Further studies are warranted to elucidate the mechanism of action.

## Introduction

Lysine demethylases catalyze the N-demethylation of histone lysine residues, and lysine-specific demethylase 1 (LSD1) specifically demethylates H3K4me1/2 and H3K9 me1/2 [1]. LSD1 also has non-histone substrates, including DNMT1, p53, STAT3, and E2F1, which play vital functions during gene expression as a part of the repressor complexes such as CoREST [2]. Several proteins including HDACs and the gene expression modulated by this complex is known to be associated with carcinogenesis. LSD1 has been reported to be overexpressed in many malignant tumors, including breast, colon, prostate, lung, gastric cancers and others [3–8]. Down regulation of LSD1 by RNAi or pharmacological inhibitors has been shown to hinder cancer progression by inducing re-expression of aberrantly silenced genes [9–12]. Several LSD1 inhibitors such as Bomedemstat, Iadademstat, etc. are in clinical trials for the treatment of AML, MPN, SCLC, etc. Vafidemstat a dual LSD1 and MAO-B targeting inhibitor is in clinical trial for the treatment of CNS disorders [13–17].

Histone deacetylases (HDACs) are a family of enzymes that typically deacetylate histone and non-histone substrates to regulate gene transcriptions that are essential for cancer cell proliferation, motility, etc. So far 18 different deacetylases have been reported that can be divided into two major classes of enzymes: Zn2+ dependent (HDAC) or NAD+ dependent (Sirtuins). Several HDAC inhibitors including Vorinostat, Belinostat, Romidepsin, and Panobinostat have been approved for the treatment of CTCL and MM and are in a clinical trial for other cancers [18–21]. These are either class I or pan-HDAC inhibitors which showed limited efficacy and tolerability as single agents and are associated with haematological toxicity, fatigue, nausea, etc. as frequent adverse effects. Therefore, these HDAC inhibitors still have limited utility due to their dose-limiting toxicity (DLT). The current hypothesis is that selective HDAC inhibitors may overcome some of the DLT observed in the clinic with the pan-HDAC inhibitors and therefore there is growing interest to develop isozyme selective HDAC inhibitors. In this regard, HDAC6 has gained a lot of attention in recent years since it is a unique HDAC in two ways: It has two catalytic deacetylase domains and its primary targets are α-Tubulin, HSP90, HSF-1, and other cytoplasmic proteins. HDAC6 isozyme belongs to the class IIB HDAC and mainly resides in the cytoplasm, but can shuttle between cytoplasm and nucleus. Several selective HDAC6 inhibitors are in the clinic for the treatment of cancer and solid tumors such as Ricolinostat, Citarinostat and KA-5407 [22–26].

Recent studies have shown that there is cross talk between LSD1 and HDACs, both of which are components of the CoREST complex, the formation of which provides an advantage to proliferation of cancer cells and survival. Accordingly, combined inhibition of LSD1 and HDAC has been shown to be more efficacious in inhibiting the growth of glioblastoma, AML and breast cancer. There is evidence to show that in certain breast cancers, HDAC6 is also part of the CoREST complex along with LSD1 and HDAC1, driving ER dependent gene transcription [27, 28]. We hypothesize that HDAC6 could be a part of CoREST and other transcriptional repressor complexes in other cancers as well where inhibiting both LSD1 and HDAC6 can lead to comprehensive inhibition of the transcription complex leading to synergistic anti-cancer activity. Besides, it is also possible that we observe better toxicity profile as compared to pan HDAC inhibitors. In this regard, we have designed and developed a novel dual LSD1-HDAC6 inhibitor with strong potency on LSD1 as well as isoform selective activity on HDAC6 and tested its anti-cancer activity in multiple haematological cancers and further carried out mechanistic studies to understand the target engagement. Results from these studies clearly demonstrate the advantage of this inhibitor over LSD1 or HDAC6 inhibitor in the treatment of a subset of cancers.

## Materials and methods

### Cell culture and compounds

All cell lines were cultured at 37°C in a humidified incubator with 5% CO2. Cell culture media and supplements were obtained from Thermo Fisher Scientific. All cell lines were obtained from ATCC (American Type Culture Collection, USA) unless stated otherwise and cultured according to instructions. JBI-097, Iadademstat and Ricolinostat were synthesised in-house by the Jubilant Drug Discovery Unit of Jubilant Biosys Ltd. The various batches of JBI-097 utilized in these studies consistently exceeded a purity of 97% as measured by Nuclear Magnetic Resonance (NMR), Liquid Chromatography- Mass Spectroscopy (LC-MS) and elemental analysis. All compounds were dissolved in dimethylsulphoxide (DMSO) as 10 milli molar stock solutions and stored -20°C. Pomalidomide and Bortezomib were purchased from selleckchem.

### Mice

All in vivo experiments were carried out at Jubilant Biosys Ltd., which has AAALAC (Association for Assessment and Accreditation of Lab Animal Care International) accreditation. Animals were quarantined for a week upon arrival and healthy animals were approved for the study.

All SCID beige and Balb/c mouse used in this study were purchased from Vivo Biotech in Hyderabad, India [under licence from Taconic]. The animals were housed in techniplast individually ventilated cages (n = 5 per cage) in a temperature-controlled environment with a 12-hour light/dark cycle. Animals were fed enough with an autoclaved commercial diet (Nutrilab Rodent Feed, spherical pellets) and autoclaved water *ad libitum*. All the animals were monitored daily by the same staff for the duration of the experiment, and were weighed three times per week. The animal studies associated with Fig 5a-5f were conducted according to the protocols approved by the Institutional Animal Care and Use Committee (IAEC/JDC/2019-174R). During the study, the care and use of animals was in accordance with the principles outlined in the Guide for the Care and Use of Laboratory Animals, 8^th^ Edition, 2010 (National Research Council). Institutional Animal care and Use Committee (IACUC) approved general humane endpoints for tumor models were applied to this study. Mice were euthanized at the completion of the study protocol by carbon dioxide overdose. Mice were sacrificed following the development of any of the following clinical signs of tumor progression: severe head tilting, ataxia, circling, lethargy and/or weight loss ≥20% of the initial weight.

### LSD1 expression and inhibition assay

Human recombinant LSD1 was expressed and purified as described below. The N-terminal truncated Human LSD1 (Isoform 1): 172a.a-852a.a containing SWIRM Domain was PCR amplified from HEK293 cDNA, sub‐cloned into a pFastbac1 vector, and expressed in insect cells carried out using Bac-to-Bac system with cleavable N-terminal 6xHis tag. Briefly, Sf9 cells in suspension culture were full-grown at 27°C in 2.8 liters baffled flasks containing a 1000 ml of SF900 II SFM medium (Gibco), shaken at 70 rpm. The cells were infected at a density of 1.2–1.5 x 10^6^ cells/mL using a multiplicity of infection (MOI) of 2. One mL samples were collected at indicated times to measure viability (Cedex Cell counter), and expression was evaluated by western blot using an antibody against the 6 His tag. 12g of cell pellet, was harvested from 2 L of culture, re-suspended (Resuspension/Binding Buffer: 20 mM HEPES pH 7.9, 300 mM NaCl, 0.5 mM TCEP, 10% Glycerol, 1X Protease inhibitor cocktail), sonicated, clarified by centrifugation. Then, the supernatant was loaded onto 5 mL Ni-affinity chromatography resin (GE Healthcare), the column was washed with the 10 column volumes (CV) of binding buffer supplemented with increasing concentrations of imidazole (5 to 35 mM). Imidazole and protein eluted in 10 CV gradient with 35–500 mM Imidazole. Then, the protein was further purified by Superdex-200 Size exclusion chromatography (GE Healthcare) (20 mM HEPES pH 7.9, 150 mM NaCl, 1.0 mM TCEP, 10% Glycerol) and the purified enzyme was used for the assay. LSD1 inhibitor tranylcypromine (TCP), was procured from Selleckchem. LSD1 enzyme, TCP, and biotinylated peptide substrate were diluted in assay buffer just before use. 2X inhibitor (10 μL, diluted in assay buffer) or assay buffer, and 5 nM enzyme were added to a 96-well plate and incubated at room temperature for 30 min. 5 μL of biotinylated histone H3K4me1 peptide (4X) was added to each well and incubated at room temperature (RT) for 1 h. Stop solution containing 300 μM TCP in 1X LANCE detection buffer from Perkin Elmer was added to the wells and incubated for 5 min at RT. Then, a detection mix containing 2 nM Eu-Ab and 50 nM ULight-Streptavidin (Perkin Elmer) in 1X LANCE detection buffer was prepared and added to the reaction mixture. This mixture was incubated for 1 hour at room temperature. Readings were taken using the Pherastar Reader in TR-FRET mode (excitation at 337 nm & emission at A-665 nm, B-620 nM). All chemicals were purchased from Sigma–Aldrich unless specified.

### HDAC isoform inhibition assay

Dose-response studies for HDAC isoforms were conducted at Reaction Biology Corporation. All assays are based on the same principle of a two-step reaction: first, the substrate with the acetylated lysine side chain is incubated with a sample containing HDAC enzyme, to produce the deacetylated products, which are then digested in the second step by the addition of the developer to produce the fluorescent signal proportional to the amount of deacetylated substrates. Recombinant proteins HDAC1, HDAC2, HDAC3, HDAC4, HDAC5, HDAC6, HDAC7, HDAC8, HDAC9, and HDAC10 were incubated with a test compound for 5min, and then the fluorophore-conjugated substrate was added to the wells and incubated for 30 min to 2 h for different HDACs. Reactions were performed in assay buffer (50 mM Tris-HCl, pH 8.0, 137 mM NaCl, 2.7 mM KCl, 1 mM MgCl2, 1 mg/mL BSA, 1% DMSO, pH 7.4) and fluorescence measurements were obtained approximately every 20 min for an hour using an Envision, Perkin-Elmer plate reader (Excitation 360 nm/Emission 460 nm). IC_50_ values and curve fits were obtained using (GraphPad Prism Software, Inc.).

### HDAC6 inhibition assay

HDAC6 inhibitory activity was determined for each compound using the Fluorogenic HDAC6 assay kit from BPS Bioscience (Catalogue No: 50077). Assays were performed according to the manufacturer’s instructions as described below. This assay involves a two-step reaction: first, the substrate with the acetylated peptide was incubated with HDAC6 enzyme, to produce the deacetylated product, which was then digested in the second step by the addition of developer (Catalogue No: 50030) to produce the fluorescent signal proportional to the amount of deacetylated substrates. Reactions were carried out in 96-well plates (Nunc low binding, black microtiter plates) and compounds were screened at 10 concentrations using 3-fold serial dilutions to generate IC_50_s. 50 μl reactions containing HDAC assay buffer (Catalogue No: 50031), 0.1 mg mL^−1^ BSA, 200 μM HDAC substrate 3, and varying concentrations of inhibitor were initiated by the addition of HDAC6 (final [HDAC6] = 35 ng) and incubated at 37°C for an hour. After incubation, the reactions were quenched by the addition of 50 μL of undiluted 2x developer and incubated at room temperature for 15 min. The fluorescence was measured using a Tecan infinite M200 reader (excitation, 355 nm; emission, 460 nm). IC_50_ values and curve fits were obtained using GraphPad Prism 5.0. (GraphPad Software, Inc.). Data are representative of at least two independent experiments.

### Thermal shift assay

The melting temperature (T_m_) of human LSD1 and HDAC8 proteins were determined by fluorescence based thermal shift assay. Reaction mixture containing 0.5 μM protein, 5X SYPRO™ Orange Protein Gel Stain (Thermo Fisher Scientific Cat: S6650), compound at 10-fold excess for hLSD1, 15-fold excess for hHDAC8. The final volume made up with respective protein buffers. All experiments were done in triplicates at 50 μL reaction volume. The samples heated from 15°C to 95°C with an increment of 1°C per 30sec using CFX96™ Real-Time PCR Detection system–C1000 Thermal Cycler (Bio-Rad). Filters custom configured to the optimal excitation (492 nm) and emission (610 nm) wavelengths for SYPRO orange dye. Protein denaturation monitored by the increase in fluorescence signal emitted by SYPRO Orange. The SYPRO Orange binds to the exposed hydrophobic regions of protein undergoing thermal denaturation and corresponding fluorescence emissions are recorded as melt curves. The temperature corresponding to the inflection point, Tm is determined by calculating the first derivative from melt curve using CFX manager 3.1 software (Bio-Rad).

### Cell treatment with compounds

Test and reference compound stocks were prepared in 100% DMSO as single use aliquots and stored at -20 °C. These stock solutions were added to culture medium to achieve the desired test concentrations. DMSO concentration was kept uniform across the plate and did not exceed 1%.

### Cell proliferation assay

The effect of JBI-097 on cell proliferation of a panel of tumor cell lines from both haematological and solid tumor origin, was assessed using Alamar Blue-based assay (Thermofischer scientific) as per the manufacturer’s instructions. Cells were seeded in NUNC 96-well tissue culture plates (Life Technologies) in 100 mL culture medium and incubated at 37°C/ 5% CO_2_ to adhere to plastic for 18–24 h. After 16–24 h, compounds were added to the cells at 10 concentrations ranging from 10–0.0005 μM prepared in 3-fold serial dilutions. Cells were incubated for 68–72 h at 37°C/ 5% CO_2_. One set of plates (designated for the 144 h read out) were centrifuged at low speed for 30 seconds, and respective compounds were replenished in fresh media and incubated for another 68–72 h at 37°C/ 5% CO_2_. At the end of incubation period, Alamar Blue™ reagent was added and incubated for 1–3 h at 37°C/ 5% CO_2_ and fluorescence was read on a Tecan M200 plate reader at 540 nm excitation, 590 nm emission wave lengths. The cellular GI_50_ value corresponds to the concentration at which 50% growth inhibition was achieved. The inhibition curve was then fitted by plotting % inhibition against log concentration in GraphPad Prism 5.0 (GraphPad Software, Inc.) and the GI_50_ value of compound was determined. Data are representative of at least two independent experiments.

### Western blot

Cells were harvested and washed twice with cold PBS after the indicated treatments. Cell pellets were lysed in 30μl of lysis buffer (50 mM Tris-HCl, pH 7.4, 150 mM NaCl, 1% Triton X-100, 0.1% SDS, 1 mM EDTA, 1 mM Na_3_VO_4_, 1 mM NaF, and protease inhibitor cocktail) for 30 min on ice. Lysates were centrifuged at 12,000 ×g for 20 min at 4°C, and the protein content in the supernatants was determined using the Bradford reagent (Bio-Rad). Equal amount of protein lysate was resolved by SDS-PAGE and transferred onto a nitrocellulose membrane (Bio-Rad). Membranes were blocked with 5% non-fat milk powder in PBS-Tween 20 buffer for 1 h, followed by incubation with primary antibody at 4°C overnight. Membranes were washed in PBST and then incubated with HRP-conjugated anti-goat or mouse anti- IgG (Cell Signalling Technology) antibody and detected using the Millipore ECL Western Blot substrate. β-actin was used as a loading control. Antibodies were obtained from the following sources: Acetyl-α- Tubulin (Cell Signalling Technology, 3971S, 1:500), β-actin (Santa Cruz Biotechnology, sc47778, 1:2000), HRP-conjugated secondary antibodies (Santa Cruz Biotechnology, 1:2000 for β-actin blots, 1:1000 for all others). Blots shown are representative of at least two independent experiments.

### Quantitative real time-PCR

Total RNA was isolated using the TRIzol (Thermo Fisher Scientific, Waltham, MA, USA) reagent according to the manufacturer’s instructions. The obtained RNA was reverse-transcribed to synthesize the complementary DNA from RNA using High Capacity cDNA Reverse Transcription Kit (Applied Biosystems, U.S.). DyNAmo ColorFlash SYBR Green qPCR Kit (Thermo Fisher Scientific) was used with cDNA and gene specific primers at a final volume of 10 μL. Real-time quantitative PCR was performed for 40 cycles of 15 s each, at 95°C and 30 s at 60°C, using a CFX96 real time machine (Bio-Rad). All reactions were performed in duplicate and the data was calculated using the delta (delta Ct) method. The relative gene expression levels for a target gene was normalized to the expression of β-actin. All the primer sequences were procured from Eurofins Genomics India Limited. Primer sequences for the analysed genes are as follows:

beta Actin F: 5′-GTGGGGTCCTGTGGTGTG-3′, beta Actin R: 5′-GAAGGGGACAGGCAGTGA-3′. Gfi1b F 5′-CCTCTTGTGCCCAGCACT-3′, Gfi1b R: 5′- CGTGAGGGGTGGAGAAGAC-3′, CD86 F: 5′- ACAGCAGAAGCAGCCAAAAT-3′, CD86 R: 5′- GAATCTTCAGAGGAGCAGCAC-3′, CD11bF: 5′-GGCATCCGCAAAGTGGTA-3′, CD11b R: 5′-GGATCTTAAAGGCATTCTTTCG-3′

### In vivo studies

To evaluate the efficacy of JBI-097 in vivo, tumor xenograft models were developed in female SCID beige mice (6–8 weeks old, Vivo Biotech, India [under the license from Taconic]) by injecting subcutaneously with 5 × 10^6^ erythroleukaemia cells (HEL92.1.7) or Multiple Myeloma cells (MM1.S) in 100 μL of serum-free medium mixed with 1:1 ratio of matrigel (BD, USA). When the tumors reached an approximate volume of 100 mm^3^, the mice were randomized into different treatment groups with a comparable average tumor volume across the groups using the standard = RAND() function in Microsoft Excel.

Mice injected with HEL92.1.7 were either treated with dosing vehicle (0.5% methylcellulose) or JBI-097 at 25 and 50 mg/kg; PO; QD. Mice with MM1.S tumors were either treated with dosing vehicle (0.5% methylcellulose), JBI-097 12.5 mg/kg; PO; QD, Pomalidomide (2.5 mg/kg, PO for 5 days per week), Bortezomib (0.5 mg/kg; Intraperitoneal IP; twice a week, BIW), the combination of JBI-097 and Pomolidamide or combination of JBI-097 and Bortezomib. For CT-26 syngeneic mouse colon cancer model female BALB/c (6–8 weeks old, Vivo Biotech, India) were used. 1x10^6^ CT26 tumor cells in 100 μL of serum-free medium mixed with 1:1 ratio of matrigel were injected s.c. into the right flank of mice. When tumors reached 70–100 mm^3^; mice were randomized into various treatment groups and dosed either with vehicle control (0.5% methylcellulose) or JBI-097 (20 mg/kg; PO; for 5days on and 2 days off), or anti-mouse PDL-1 antibody (BioXcell) (i.p. at 100 μg per mice Q4D). During the experiment, body weights were measured 2–3 times a week and tumor volume was measured using callipers every other day in three dimensions. Tumor volume was calculated from the pi-based ellipsoid volume formula π/6*length*width*height. Mice were culled when the tumor volume reached either the ethical limit of 2000 mm^3^ or if the tumor was ulcerated. The percentage change in tumor volume from baseline was used to assess the response to treatment. Tumor measurements were performed in a blinded manner throughout the study.

## Results

### Biochemical potency of JBI-097

**Fig 1:**
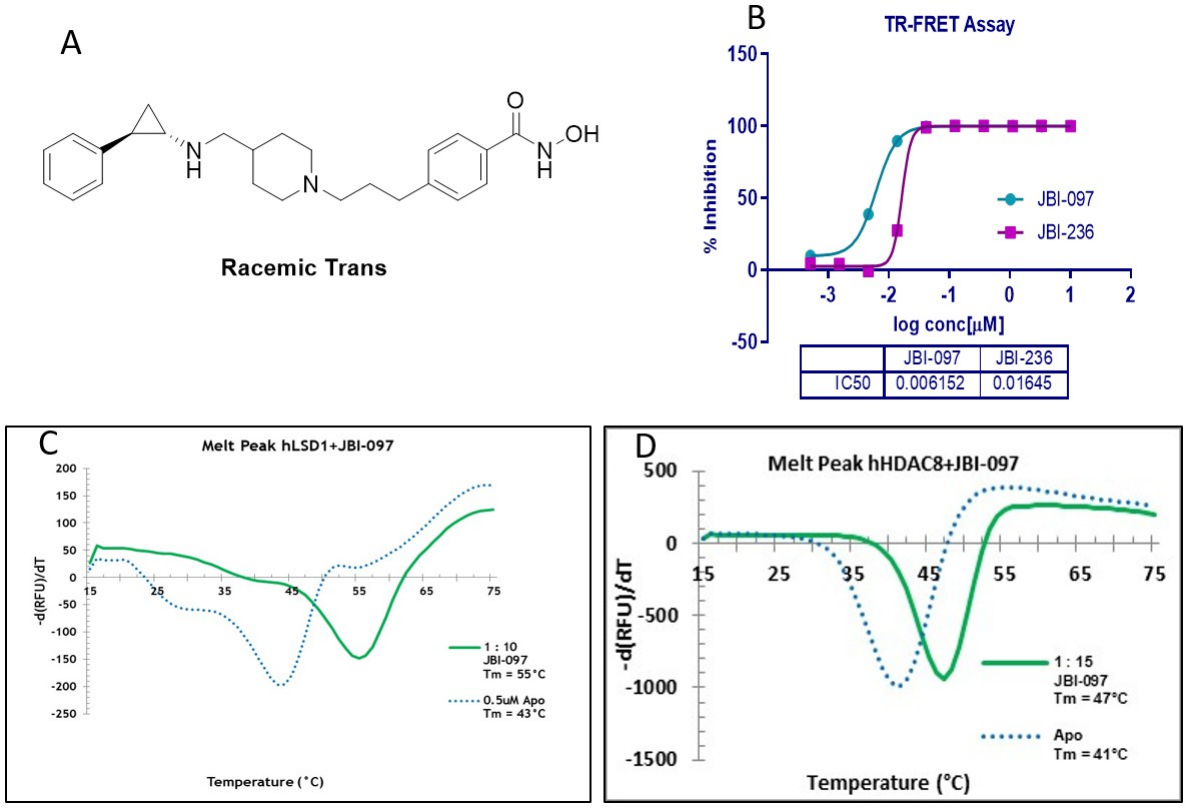
Structure, biochemical activity and melt curve of JBI-097. **(1A)** Chemical Structure of JBI-097. (**1B)** JBI-097 potently inhibited LSD1 in TR-FRET Assay. The results shown are representative from two independent experiments. (**1C and 1D)** Derivative melt curves of 1C. LSD1 and 1D. HDAC8 in the presence of JBI-097 or DMSO. Experiments were done in triplicates.

Compounds targeting LSD1 and HDAC6 were designed using both SBDD and rationale approach with a clear understanding of specific pharmacophore requirements of the LSD1 inhibition and HDAC inhibition. Several compounds hypothesized to have LSD1 and HDAC6 inhibitory activity were synthesized and profiled in LSD1 and HDAC6 enzyme activity assay. The structure of one of the leads JBI-097 is depicted in the Fig 1A. We recently published the results of the SAR analysis, PK profile and synthetic methodology of JBI-097 and other various compounds [29]. Here in we describe the biological characterization of the lead molecule JBI-097.

JBI-097 showed a strong potency in inhibiting LSD1 and HDAC6 enzymatic activities with an IC_50_ of 6 (Fig 1B) and 48 nM, respectively. JBI-097 was screened against a panel of HDACs comprising of HDAC1 to HDAC10 (Table 1) (Reaction Biology corp., Malvern, USA) where it showed strongest activity against HDAC6 with an IC_50_ of 48 nM, followed by HDAC8 (IC_50_: 71 nM). Against the remaining HDACs it showed an IC_50_ of about 1.1 μM to >10 μM with ~48 to ~70-fold selectivity against HDAC1 as compared to HDAC8 and HDAC6 IC_50_s, respectively. Therefore, it was clear that JBI-097 was a HDAC6/8 selective inhibitor. Ricolinostat, another reported HDAC6 inhibitor that is in clinical trials also showed strongest potency against HDAC6, and its potency on class I HDACs was in the range of 50 nM.

**Table 1:** HDAC Isoform Selectivity of JBI-097.

HDAC Isoform selectivity	JBI-097 IC50 nM^b^	Ricolinostat, IC50, nM^a^
HDAC1	3400	48
HDAC2	3950	51
HDAC3	7470	7000
HDAC5	1140	5000
HDAC6	48	5
HDAC7	3580	1400
HDAC8	71	100
HDAC9	3430	>10000
HDAC10	>10,000	ND

^a^Data from literature [30]

^b^Generated at Reaction Biology Corporation

For further confirmation of JBI-097 binding to LSD1, thermal shift assays were carried out by SYPRO-Orange dye incubation followed by PCR to assess the melting temperature (T_m_). T_m_ of full length LSD1 protein in the absence of compound was 43°C whereas when incubated with JBI-097 it shifted to 55°C suggesting that binding of the compound stabilized the protein (Fig 1C). Similarly, when JBI-097 was incubated with full length HDAC8, clear shift in Tm (41°C to 47°C) was observed (Fig 1D).

### Anti-cancer activity

JBI-097 when tested in a panel of non-small lung cancer, melanoma, and hepatocellular carcinoma tumor cell lines for 6 days, showed a strong anti-proliferative activity with EC50 values ranging from 1.06 to 8.09 μM (Table 2). Similarly, JBI-097 inhibited the proliferation of haematological cancers like acute myeloid leukaemia (AML), chronic myeloid leukaemia (CML), erythroleukaemia, and multiple myeloma with stronger potency with EC50 values ranging from 0.002 μM to 1.5 μM (Table 2).

**Table 2:** Anti-proliferative activity of JBI-097 in various cancer cell lines.

Cancer Type	Cell line	JBI-097 EC_50_ μM, 144hr
Multiple Myeloma	MM1.S	0.002
	MM1.R	0.029
Leukemia	MV4-11	0.002
	OCI-AML3	0.030
	HL60	1.500
	HL60-MX1	0.034
	HEL 92.1.7	0.049
	THP-1	0.226
SCLC	H1341	3.256
	H2171	2.700
	H510A	1.060
Melanoma	HT144	3.140
	RPMI-7591	8.090
	WM-266-4	6.831
	Colo-289	2.185
	SKMeI2	1.828
	G362	7.637
Hepatocellular Carcinoma	Hep-G2	1.100
	Hep3B2	1.300

**Fig 2:**
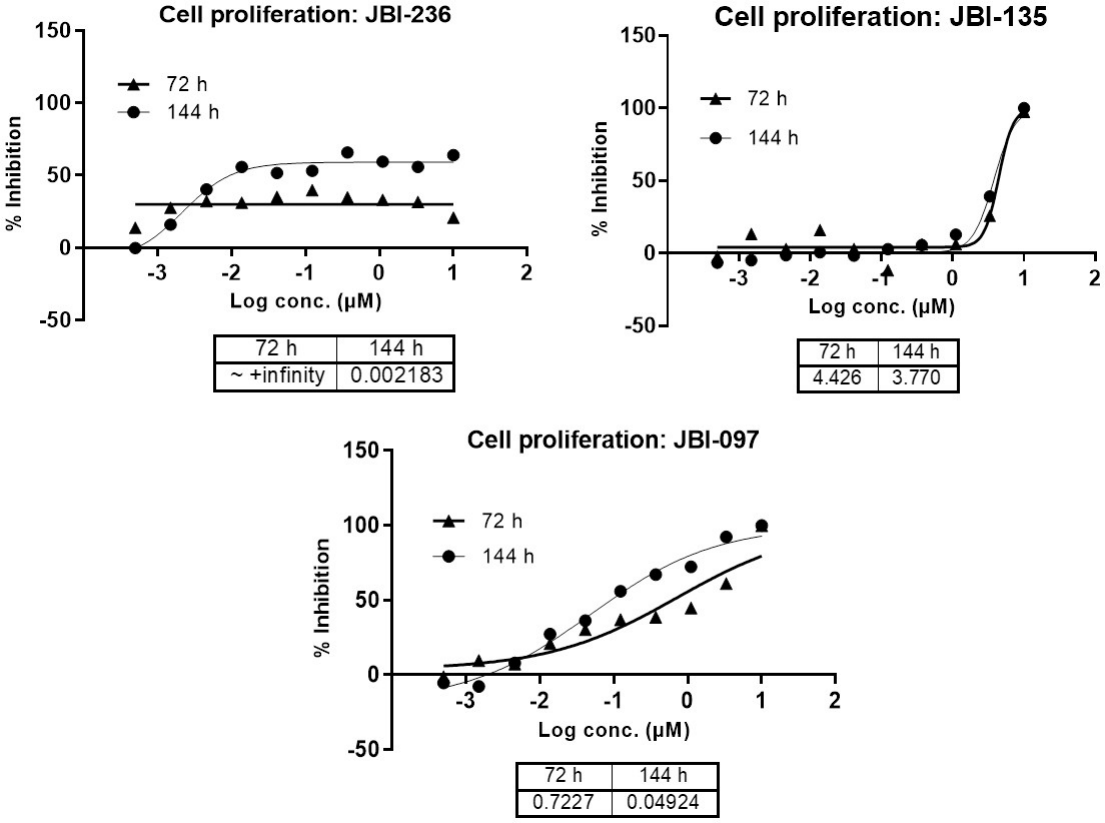
Anti-proliferative activity of JBI-097 in human erythroleukemia HEL 92.1.7 cells. Cell proliferation was evaluated using a Alamar Blue assay. Human cell lines (HEL 92.1.7; density, 5 and 10 × 10^3^ for 3 days and 6 days; respectively) were incubated with or without the indicated concentrations of JBI-097, Ricolinostat and Iadademstat for 72 and 144 h. The results shown are representative from two independent experiments.

To ascertain that both LSD1 and HDAC6 mechanisms are operating, 3 day and 6-day proliferation assays were carried out with JBI-097 along with LSD1 (Iadademstat) and HDAC6 (Ricolinostat) selective inhibitors. With the dual inhibitor JBI-097, in HEL92.1.7 erythroleukaemia cells, anti-proliferative activity was observed even at 3 days of incubation with an EC50 of 0.722 μM similar to the HDAC6 inhibitor, suggesting that the initial activity observed could be through HDAC6 inhibition. LSD1 selective inhibitor did not show any measurable anti-proliferative activity at 72 h but showed a strong anti-proliferative activity at 6 days of incubation; but interestingly, cell growth inhibition saturated at 50–60% and complete inhibition was not achieved even at a very high concentration of 10 μM. With HDAC6 inhibitor Ricolinostat, comparable anti-proliferative activity was observed both at 72 and 144 h of treatment with an EC50 of 4.4 and 3.7 μM, respectively. JBI-097 on the other hand showed a strong anti-proliferative activity with 100% cell growth inhibition on day 6 with an EC50 of 0.049 μM. This strong increase in potency at 6 days of treatment suggests that the LSD1 mechanism is operational in the dual inhibitor (Fig 2). These results clearly suggest that both LSD1 and HDAC6 mechanisms are targeted by the dual inhibitor.

### Biomarker studies

**Fig 3:**
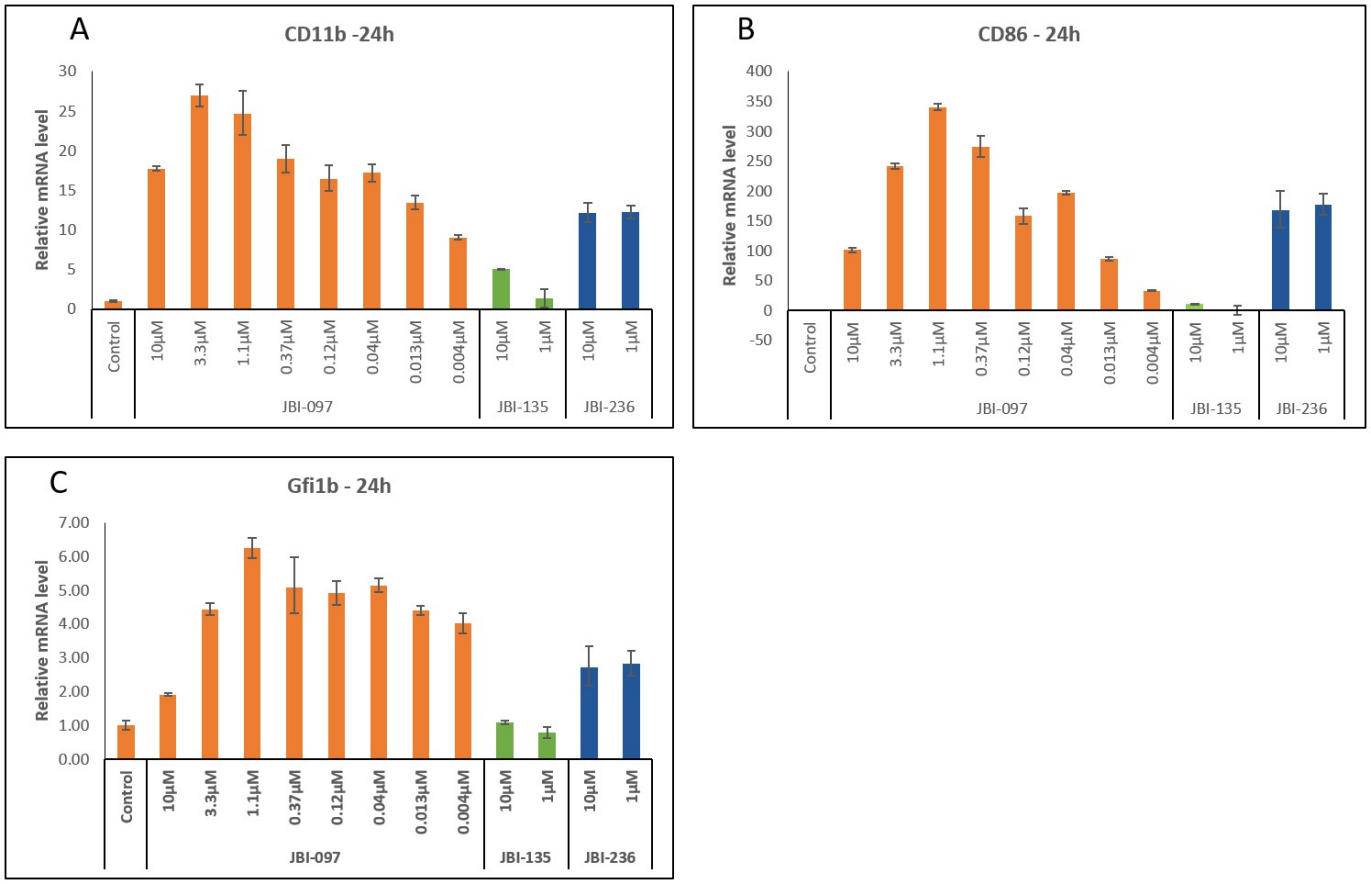
JBI-097 effect on the modulation of differentiation markers in HEL 92.1.7 cells. **(3A)** Effect of JBI-097 on CD11b modulation in HEL 92.1.7 Cells. Cells were treated with 8 concentrations of JBI-097, 10 and 1 μM of JBI-135 (Ricolinostat) and JBI-236 (Iadademstat) for 24h and the mRNA level of CD11b was estimated by RT-qPCR. Error bars indicate mean ± S.E, (**3B)** Effect of JBI-097 on CD86 modulation in HEL 92.1.7 Cells. Cells were treated with 8 point CRC of JBI-097, 10 and 1 μM of JBI-135 (Ricolinostat) and JBI-236 (Iadademstat) for 24h and the mRNA level of CD86 was estimated by RT-q PCR. Error bars indicate mean ± S.E, (**3C)** Effect of JBI-097 on Gfi1b modulation in HEL 92.1.7 Cells. Cells were treated with 8 point CRC of JBI-097, 10 and 1 μM of JBI-135 (Ricolinostat) and JBI-236 (Iadademstat) for 24h and the mRNA level of Gfi1b was estimated by RT-q PCR. Error bars indicate mean ± S.E.

**Fig 4:**
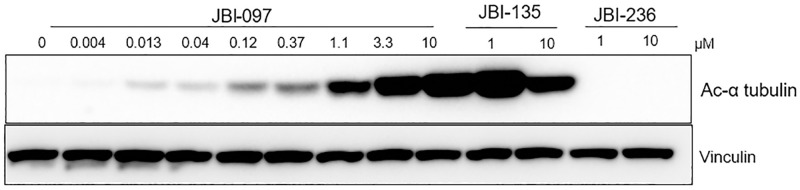
JBI-097 inhibition increased tubulin acetylation in HEL92.1.7 cells. HEL 92.1.7 cells were cultured with vehicle control, JBI-097 (3fold—8point dose response from 10 μM), Ricolinostat (10, 1 μM) and Iadademstat (10, 1 μM) for 3 h. Whole-cell lysates were subjected to immunoblotting with the indicated antibodies. Vinculin was used as loading control.

We further carried out biomarker studies to understand the target engagement of the dual inhibitor on both the targets. Erythroleukemia cell line, HEL92.1.7 was treated for different durations (3–48 h) with the dual inhibitor and differentiation markers GFI1b, CD11b and CD86 were assessed. JBI-097 showed a strong and sustained up-regulation of these markers at all the time points tested. For each marker maximum modulation was observed at 24 h. Also, for some of the markers, slight decrease was observed at higher concentrations (Fig 3A–3C). Similarly, effect of JBI-097 on acetylation of α-Tubulin was assessed at various time points where a time and concentration dependent increase was observed from 3 to 48 h and representative blots of 3 h is shown in (Fig 4).

### Efficacy study

**Fig 5:**
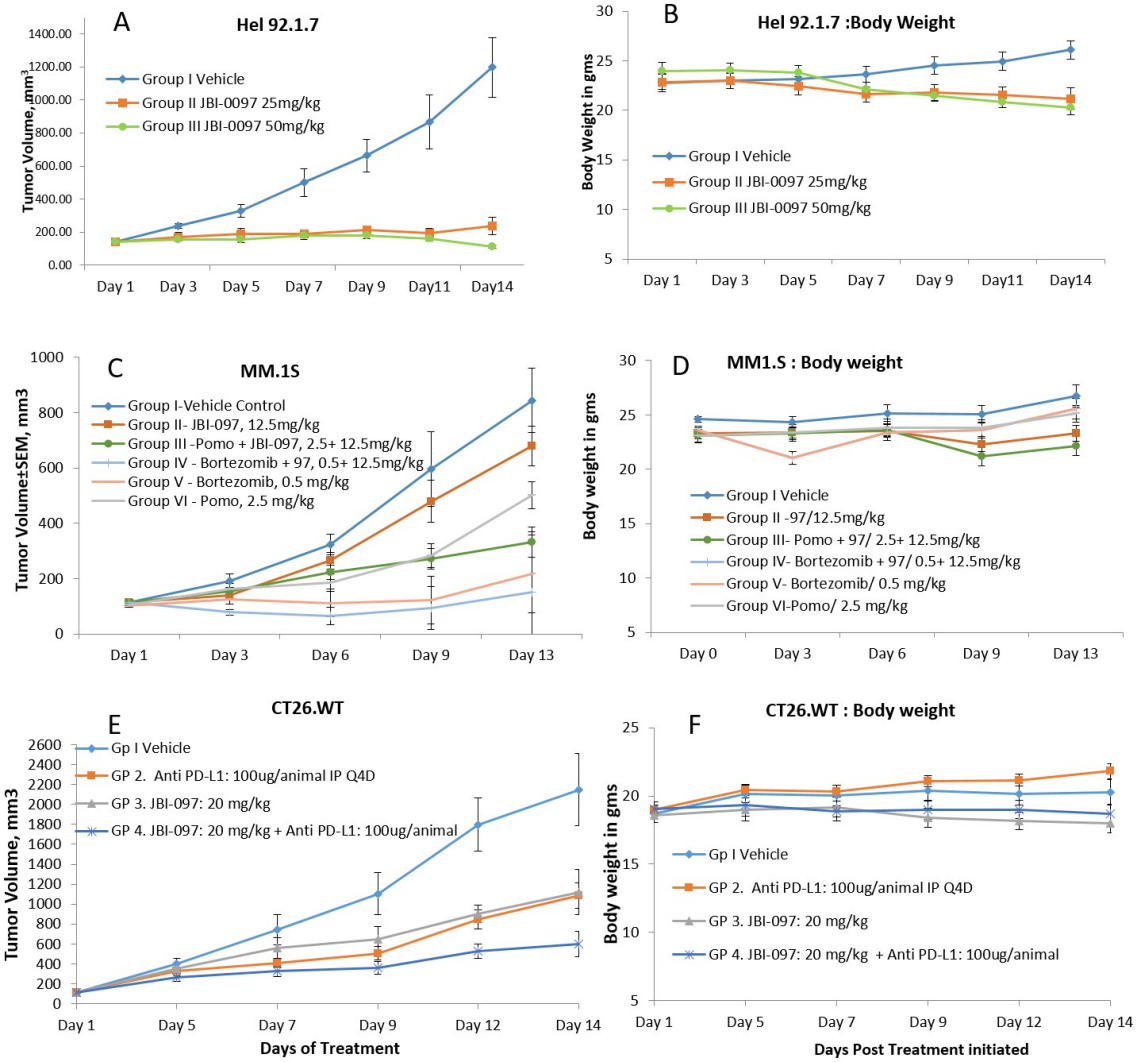
Efficacious of JBI-097 in multiple Xenograft models. **5A:** SCID mice were injected s.c. with 5 × 10^6^ HEL 92.1.7 cells and were treated with vehicle, JBI-097 (25 and 50 mg/kg, po, QD), for 14 d.; n = 6 mice per group. **5B**: HEL92.1.7 xenograft: Body weight monitored from Day1 to Day14; n = 6 mice per group. **5C:** SCID mice were injected s.c. with 5 × 10^6^ MM1.S cells and were treated with vehicle, JBI-097 (12.5 mg/kg, po), Bortezomib (0.5 mg/kg, IP, BIW), Pomalidomide (2.5mg/kg, po, 5x/week), or Bortezomib + JBI-097 or Pomalidomide + JBI-097 for 13 d.; n = 7 mice per group. **5D**: MM1.S xenograft: Body weight monitored from Day1 to Day13; n = 7 mice per group. **5E:** BALB/c mice were injected s.c. with 1 × 10^6^ CT-26 cells and were treated with vehicle, JBI-097 (20 mg/kg, po), Anti PD-L1 (100μg/animal, IP, Q4D), or Anti PD-L1 + JBI-097 for 14 d.; n = 7 mice per group. **5F**: CT-26 syngeneic model: Body weight data from Day1 to Day14; n = 7 mice per group. All data are represented as mean ± SE. In all the studies tumor volume was measured and calculated versus time (days).

The anti-tumor activity of JBI-097 in vivo was examined in xenograft mouse models of erythroleukaemia (HEL92.1.7) cells or multiple myeloma cells (MM1.S). When HEL92.1.7 xenograft tumors, reached a size of ~100 ± 10 mm^3^, mice were randomized into various groups (n = 6) including vehicle (0.5% methyl cellulose, PO), JBI-097 25 mg/kg, and JBI-097 50 mg/kg, PO, QD. Mice were dosed for 14 days or until the endpoint tumor volume of 1200 ± 100 mm^3^ was achieved. As shown in Fig 5A, JBI-097 suppressed the growth of HEL92.1.7 tumors in a dose-dependent manner compared to the control treatment. The % tumor growth inhibition (TGI) of JBI-097 was 76% and 91%, for 25 and 50 mg/kg groups, respectively. Treatment at all dose levels was well tolerated with no mortality; body weight changes were within acceptable limit and a loss of around 5–10% was observed in 2 animals from 50 mg/kg group (Fig 5B).

We further investigated the efficacy of JBI-097 in multiple myeloma MM1.s xenograft model (n = 7) in combination with Bortezomib (BTZ) and Pomalidomide. As shown in Fig 5C, while the JBI-097 as a single agent showed TGI of 23% at 12.5 mg/Kg dose, the combination of JBI-097 with BTZ led to a more potent reduction in tumor volume, with a TGI of 82%, similarly, JBI-097 showed a TGI of 60% in combination with pomalidomide. Bortezomib treatment did cause some toxicity as observed by body weight reduction and mortality of 1 mouse (1/7) Pomalidamide alone as well as in combination with JBI-097 was well tolerated throughout the study (Fig 5D).

We also further investigated the efficacy of JBI-097 as a single agent and in combination with anti-PD-L1 antibody in inhibiting the tumor growth in CT-26 mouse colon cancer derived syngeneic model (n = 7). As shown in Fig 5E, JBI-097 and anti-PD-L1 alone showed a TGI of 50 and 52%, respectively while the combination of JBI-097 with anti-PD-L1 led to a stronger TGI of 76%. All the treatment groups were well tolerated with no significant body weight loss or mortality (Fig 5F).

## Discussion

Despite the improvements in treatments of cancers during the past decades, resistance to chemotherapeutic agents and/or targeted drugs is still a major problem in cancer therapy and is responsible for most relapses [31]. Due to the emergence of resistance, therapeutic agents targeting a single protein/mechanism become poorly effective. Several studies have shown that combination treatment strategies help to improve efficacy and prolong or prevent resistance [32, 33]. An alternative and recent strategy that could be applied is the dual-target approach by one molecule which inhibits two enzymes simultaneously and offers an opportunity for poly pharmacology leading to synergistic efficacy. Recently Duan YC, et al, demonstrated a bivalent approach of targeting LSD1 and HDACs. Although the mechanism of dual-action inhibitors was not well-established, these studies demonstrated the advantage and benefits of this strategy [34]. Previously, we reported JBI-097 as a potent and highly selective LSD1/HDAC6 inhibitor and in the present study, we further characterized the molecule to understand the dual action. Our results indicated that JBI-097 biochemically is highly potent for LSD1 and HDAC6 and selective against other HDACs. Treatment with JBI-097 resulted in decreased viability of hematological and solid tumor cells in a dose- and time-dependent manner. Previously published data using small molecule inhibitor and knockdown studies have suggested that inhibition of LSD1 promotes upregulation of two myeloid differentiation markers (CD11b and CD86) in leukemic cells [35, 36] which is consistent with our results. JBI-097 treatment consistently increased differentiation/myeloid maturation markers CD11b and CD86 indicating inhibition of LSD1. These results suggest that the anti-proliferative effect of JBI-097 is at least in part by differentiation of the HEL92.1.7 cells. Consistent with our findings, others groups have reported that pharmacological inhibition of LSD1 increases C11b and CD86 in leukemic cells. Interestingly, Fang J et al have shown that LSD1 inhibition did not alter the expression of global H3K4me2 levels, whereas it induces expression of CD11b and CD86 through increasing H3K4me2 levels on the proximal promoter region of those two genes, suggesting that differentiation effect of LSD1 inhibition depends on its histone demethylase activity on these specific target genes [37, 38]. In contrast, Feng and Fiskus’s groups reported that pharmacological inhibition of LSD1 globally increases H3K4 methylation in some specific AML cells, which suggests it is a cell context-dependent pattern [39, 40]. In hematopoietic cells, LSD1 has demonstrated that the catalytic domain of LSD1 is spacious–containing the FAD cofactor–and can bind to proteins and histone tails. Of these proteins that bind in the catalytic pocket of LSD1 are transcription factors, GFI1 and GFI1B, where the functionality of the interaction leads to the proliferation of normal and malignant hematopoietic cells [41]. Maiques et al demonstrated that LSD1 inhibition displaces GFI1/1B and CoREST proteins with small molecule inhibitors, which induces differentiation of hematopoietic cells [42]. Recently, Y. Ishikawa et al had shown with T-3775440 disrupted the LSD1–GFI1B interaction, that successively evoked the transcriptional derepression of GFI1B target genes and consequent trans differentiation thereby causing antileukemic efficacy [43]. Similarly, our results had shown the modulation of GFI1B upon treatment with JBI-097 which suggests the disruption between the LSD1-GFI1B interaction.

Several reports highlight the cross-talk between lysine specific demethylase 1 (LSD1) and histone deacetylases (HDACs) which modulate the expression of multiple disease-specific genes as part of repressor complexes, including CoREST which suggests that the activity of LSD1 may be influenced by inhibiting HDAC and vice versa and combined targeting of HDACs and LSD1 might be superior when compared to individual inhibition [44]. Vasilatos et al. reported that in human breast cancer cells the antitumor activity of HDAC inhibitors was mediated by the crosstalk between LSD1 and histone deacetylases [45]. One such compound which has been reported to target the CoREST complex is Corin, and its mechanism of dual targeting of LSD1 and HDAC1 in the CoREST complex seems to contribute to its enhanced cellular pharmacology in melanoma. This dual inhibition by Corin provides comprehensive targeting of the CoREST complex and overcomes the substantial regulatory challenge of advancing two separate compounds [46].

Although class I HDAC inhibition has been well studied, dose-limiting toxicities related to these inhibitors remain a challenge in the clinic. On the other hand, many groups are focusing on developing inhibitors selective for HDAC isoforms due to their interaction with proteins involved in cell growth, apoptosis, migration, protein degradation, etc. Further, selective HDAC6 inhibition also could circumvent dose-limiting toxicity related to pan-HDAC and class I HDAC inhibitors [47]. Among HDACs, HDAC6 is considered an important therapeutic target that brings in increased sensitivity of transformed cells to certain anticancer agents. Also, knockout (KO) studies in mice have established that HDAC6 KO mice developed normally [48]. Our results showed that JBI-097 was highly potent for HDAC6 with high selectivity against other HDACs, except for HDAC8. Accordingly, treatment of cancer cells with JBI-097 resulted in a strong and dose-dependent increase in acetylation of alpha-tubulin, a selective cellular substrate for HDAC6. We have demonstrated the dual inhibition of the targets not only based on target-specific biomarkers but also based on the cell proliferation studies where a stronger anti-proliferative response was obtained only after prolonged treatment in LSD1 sensitive cells, which is typically seen with LSD1 inhibition. Accordingly, treatment of cancer cells with JBI-097 resulted in a stronger and complete inhibition of cell proliferation as compared to LSD1 or HDAC6 specific inhibitors, clearly demonstrating the advantage of dual inhibition. In addition to HDAC6 inhibitory activity, JBI-097 also inhibits HDAC8. While some selective substrates such as structural maintenance of chromosomes 1 and 3 (SMC1 and 3) are known for HDAC8, biological outcomes with selective HDAC8 inhibition have not been well understood. Modulation of cell cycle has been reported with selective HDAC8 inhibition in malignant peripheral nerve sheath tumors [49], while others claim that HDAC8 inhibition leads to enhanced immune infiltration [50]. Multiple non-histone acetylation targets have also been identified for HDAC8 [49]. Therefore, additional studies need to be conducted to understand the potential contribution of HDAC8 inhibition in the anti-cancer effect of JBI-097, that are beyond the scope of the manuscript.

We also evaluated the anti-tumor efficacy of JBI-097 in xenograft-based in vivo studies. In these studies, JBI-097 significantly inhibited tumor growth and the combination treatment with standard of care (SoC) resulted in further stronger tumor growth inhibition. Notably, all the treatments were tolerated well as observed by the body weights of mice. Earlier, we have reported [29] that JBI-097 showed a strong single-agent anti-tumor effect in multiple myeloma (MM). Till today MM remains an unmet need with high relapse rate, which requires additional therapeutic choices, together with multidrug-combinations that induce tumor regression with less resistance and low toxicity. Bortezomib has been approved by the US Food and Drug Administration (FDA) as an effective therapy for multiple myeloma; however, prolonged treatment can be associated with toxicity and drug resistance [51]. Huang et al. reported that co-treatment with a unique selective-HDAC6 inhibitor, MPT0G413, and the bortezomib showed synergistic inhibition of multiple myeloma tumor cell viability [52]. Moreover, clinical trial studies on MM have shown that pomalidomide is an efficient drug to overcome resistance to lenalidomide and thalidomide as well as to bortezomib [53]. In the present study, we have evaluated the combination of JBI-097 with bortezomib and pomalidomide in MM and the results clearly showed a stronger and complete tumor growth inhibition of the triple combination when compared to single agents.

In addition to haematological cancer, we wanted to assess the effect of the dual inhibitor in solid tumors as well where combination approaches are essential. Since PD-1/PD-L1 based checkpoint therapies have come into mainframe therapies any strategy to enhance the effect of these checkpoint inhibitors will be highly valuable in the clinic. In this regard, activating the immune response to the tumor and/or overcoming the immunosuppressive environment of the tumor has shown to be beneficial in augmenting the response as well as overcoming the resistance to checkpoint inhibitors. Recent reports have shown that HDAC6 plays an essential role as a checkpoint regulator in melanoma cells [54]. Use of panHDACi, such as panobinostat, is known to increase the expression of the immunosuppressive proteins such as programmed death ligand-1 (PD-L1) and programmed death ligand-2 (PD-L2) on the cell surface of tumor cells [55]. Also, Ye qin et al had shown that LSD1 inhibition reactivates key immune regulators and cytotoxic T cells attracting chemokines that make TNBC sensitive to immune checkpoint blocking antibodies [56]. Genetic ablation or pharmacological inhibition of LSD1 has been shown to enhance tumor immunogenicity and T cell infiltration and also overcomes resistance to anti-PD1 inhibitors [57]. Lienlaf et al had demonstrated a role of HDAC6 in the regulation of PD-L1 in melanoma [58]. Recently, Arghya Ray’s studies also demonstrated that combination of HDAC6 selective inhibitor ACY-241 and anti-PD-L1 Ab restore immune function and enhance MM cytotoxicity [59].

Studies have shown that the 4T1 cell model treated with HDACi significantly increased PD-L1 expression, and tri-therapies with 5-Aza or entinostat showed complete remission of tumors. Also, in most of these models single-agent checkpoint inhibition has been shown to be only moderately efficacious suggesting that appropriate combination approaches are essential to achieve complete tumor growth inhibition [60, 61]. In the present study, we investigated whether JBI-097 could augment antitumor immune responses and improve the therapeutic efficacy of immune checkpoint blocking antibodies. Our findings indicate that the combined use of JBI-097 with anti-PD-L1 antibody effectively enhances the therapeutic efficacy. In conclusion, both in vitro and in vivo studies strongly suggest that JBI-097 acts via the simultaneous inhibition of the LSD1 and HDAC6 pathways, to inhibit proliferation, enhance differentiation, and consequent tumor inhibition.

## Conclusion

Our findings suggest that comprehensively targeting the LSD1 and HDAC6 complex can enhance therapeutic response when compared to single agents. However, further genomic and mechanistic studies will be needed to have a clear understanding of how dual targeting of the LSD1 and HDAC6 leads to enhanced potency and efficacy in sensitive cells. Regardless, our results substantiate that such targeting can offer superior anti-tumor efficacy in malignancies that are especially sensitive to LSD1 inhibitors such as erythroleukaemia, acute myeloid leukemia as well as in other cancers such as multiple myeloma. Further, JBI-097 could also be used to safely enhance the efficacy of checkpoint therapies in solid tumors. Our studies using JBI-097 demonstrated a significant improvement in the anti-tumor effects of anti-PD-L1 immune checkpoint blockade therapy. Further studies are in progress to understand the molecular mechanism behind enhanced efficacy of the dual inhibitor. Thus, concomitant targeting of epigenetic modifiers LSD1 and HDAC6 using this LSD1/HDAC6 dual inhibitor represents a promising new strategy for the treatment of cancer that warrants further investigation.

## Supporting information

S1 File Raw images: Original full western blots corresponding to Fig 4. HEL 92.1.7 cells were cultured and treated with vehicle control, JBI-097 (3fold—8point dose response from 10 μM), JBI-135 (10, 1 μM) and JBI-236 (10, 1 μM) for 3 h. Whole-cell lysates were subjected to immunoblotting with the acetyl-α-tubulin (top panels). Vinculin was used as loading control (bottom panels). Arrows indicate the bands representing Acetyl-α-tubulin and Vinculin.
(PDF)

S1 Checklist (PDF)
