# Supplementary material for: Novel dual LSD1/HDAC6 inhibitor for the treatment of cancer
Source: PLoS One. 2023 Jan 3;18(1):e0279063. doi: 10.1371/journal.pone.0279063 (PMC9810167; doi:10.1371/journal.pone.0279063)
Supplement: S1 File — HEL 92.1.7 cells were cultured and treated with vehicle control, JBI-097 (3fold—8point dose response from 10 μM), JBI-135 (10, 1 μM) and JBI-236 (10, 1 μM) for 3 h. Whole-cell lysates were subjected to immunoblotting with the acetyl-α-tubulin (top panels). Vinculin was used as loading control (bottom panels). Arrows indicate the bands representing Acetyl-α-tubulin and Vinculin. (PDF) [file pone.0279063.s001.pdf]

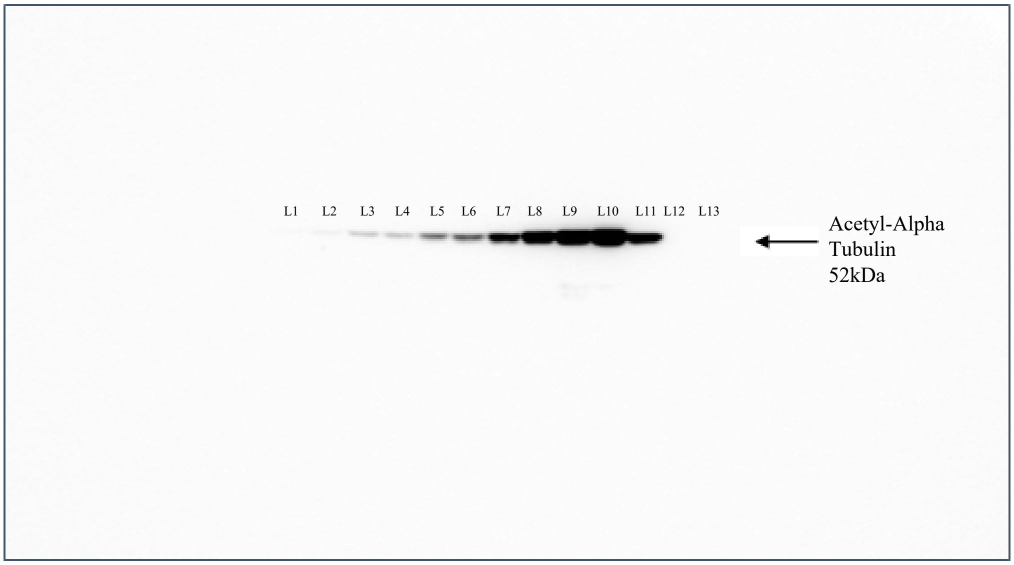

Acetyl-Alpha Tubulin		
L1	Control	
L2	0.0046	JBI-097 (µM)
L3	0.0137	
L4	0.0412	
L5	0.1235	
L6	0.3704	
L7	1.1111	
L8	3.3333	
L9	10	
L10	1	JBI-135 (µM)
L11	10	
L12	1	JBI-236 (µM)
L13	10	

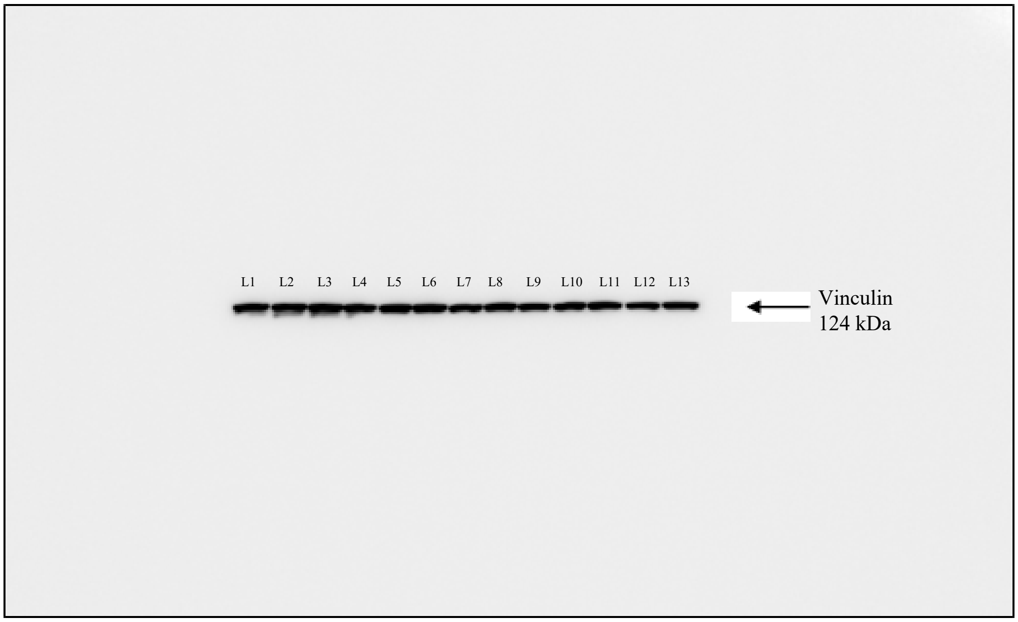

Vinculin		
L1	Control	
L2	0.0046	JBI-097 (µM)
L3	0.0137	
L4	0.0412	
L5	0.1235	
L6	0.3704	
L7	1.1111	
L8	3.3333	
L9	10	
L10	1	JBI-135 (µM)
L11	10	
L12	1	JBI-236 (µM)
L13	10	
